# Neurochemical Features of Rem Sleep Behaviour Disorder

**DOI:** 10.3390/jpm11090880

**Published:** 2021-08-31

**Authors:** Félix Javier Jiménez-Jiménez, Hortensia Alonso-Navarro, Elena García-Martín, José A. G. Agúndez

**Affiliations:** 1 Section of Neurology, Hospital Universitario del Sureste, Arganda del Rey, C/Marroquina 14, 3 B, E28030 Madrid, Spain; hortalon@yahoo.es; 2 UNEx, ARADyAL, Instituto de Salud Carlos III, University Institute of Molecular Pathology, E10071 Cáceres, Spain; elenag@unex.es (E.G.-M.); jagundez@unex.es (J.A.G.A.)

**Keywords:** REM sleep behavior disorder, neurochemistry, neurotransmitters, dopaminergic dysfunction, noradrenalin, acetyl-choline, synucleopathies

## Abstract

Dopaminergic deficiency, shown by many studies using functional neuroimaging with Single Photon Emission Computerized Tomography (SPECT) and Positron Emission Tomography (PET), is the most consistent neurochemical feature of rapid eye movement (REM) sleep behaviour disorder (RBD) and, together with transcranial ultrasonography, and determination of alpha-synuclein in certain tissues, should be considered as a reliable marker for the phenoconversion of idiopathic RBD (iRBD) to a synucleopathy (Parkinson’s disease –PD- or Lewy body dementia -LBD). The possible role in the pathogenesis of RBD of other neurotransmitters such as noradrenaline, acetylcholine, and excitatory and inhibitory neurotransmitters; hormones such as melatonin, and proinflammatory factors have also been suggested by recent reports. In general, brain perfusion and brain glucose metabolism studies have shown patterns resembling partially those of PD and LBD. Finally, the results of structural and functional MRI suggest the presence of structural changes in deep gray matter nuclei, cortical gray matter atrophy, and alterations in the functional connectivity within the basal ganglia, the cortico-striatal, and the cortico-cortical networks, but they should be considered as preliminary.

## 1. Introduction

In 1986, Schenk et al. [1,2] described a new type of parasomnia in patients (most of them men) with several neurological diseases, resembling previous findings in studies in cats with pontine tegmental lesions, that consisted in abnormal behaviors during rapid eye movement (REM) sleep such as “stereotypical hand motions, reaching and searching gestures, punches, kicks, and verified dream movements”. Polysomnography (PSG) showed variable loss of chin atonia, increased REM limb-twitch activity, increased REM ocular activity, and density and increased duration of stage 3–4 slow-wave sleep. Clonazepam and desipramine improved considerably these symptoms. This disorder was designated as “REM sleep behaviour disorder” (RBD). A review of diagnostic tools for RBD including 58 studies concluded that sleep history might be sufficient for the diagnosis of RBD, although PSG study should be necessary for a definitive diagnosis, while the value of objective measurements including visual electromyographic (EMG) scoring methods, actigraphy, cardiac ^123^I-metaiodobenzylguanidine (^123^I-MIBG) scintigraphy, and behavioral classification and video analysis was not established [3].

Olson et al. [4] described a large series (93 patients, 87% men) of patients with RBD and found the presence of neurological disorders in 57% of them (all but 14% of them with Parkinson’s disease –PD- dementia without parkinsonism, and multiple system atrophy -MSA), and described that RBD preceded parkinsonism in 52% of PD patients (although the first description of RBD preceding PD was done by Tan & Salgado [5]). In general, conventional neuroimaging showed non-specific alterations, and in 87% of patients, RBD improved totally or partially with clonazepam. Other group described that 45% of 44 patients diagnosed with RBD developed a neurological disorder (mainly PD and Lewy Body dementia—LBD) after a mean of 11.5 years from the reported onset of RBD and a mean follow-up of 5.1 years from the diagnosis of idiopathic RBD (iRBD) [6], and 82% of them (16 with PD, 14 with LBD, 1 with MSA, and 5 with mild cognitive impairment) at 14 years of follow-up [7].

On the other hand, Boeve et al. [8], in a series of 398 patients with parkinsonism and/or cognitive impairment, described a significantly higher frequency of probable RBD and PSG confirmed RBD in patients with synucleopathies (PD, LBD, or MSA) compared with those without synucleopathies (progressive supranuclear palsy –PSP-, corticobasal degeneration –CBD-, frontotemporal dementia –FTD-, Alzheimer’s disease –AD-, mild cognitive impairment –MCI-, primary progressive aphasia –PPA- or posterior cortical atrophy –PCA).

From the etiological point of view, RBD is usually classified as “idiopathic” (iRBD, when at the time of diagnosis there is no evidence of diagnosed neurological disease) or secondary. Secondary RBD is usually related to a previously diagnosed neurodegenerative disease, narcolepsy, autoimmune disease, or induced by drugs [9,10], but also related to structural lesions affecting the pons, medulla, or limbic system [11,12,13]. However, as it should be discussed later, many of the patients diagnosed with iRBD, after a long-term follow up, develop neurodegenerative diseases, mainly PD, LBD, or MSA.

A recent systematic review and meta-analysis described a pooled prevalence of defined iRBD of 0.68% (95% confidence intervals –CI- = 0.38–1.05%) in 5 studies without significant heterogeneity, and a pooled prevalence of probable iRBD of 5.65 (95% CI = 4.29–7.18%) in 14 studies with significant heterogeneity among them [14].

Regarding the etiology of iRBD, only a few studies addressed the possible role of genetic and environmental factors:

(1) A first study identified a hexanucleotide repeat expansion in the *C9orf72-SMCR8 complex subunit* (*C9orf72*) gene (chromosome 9p21.2; gene ID 203228, MIM 614260; this gene encodes a protein with an important role in the regulation of endosomal trafficking), which has been related with familial amyotrophic lateral sclerosis and FTD dementia, in 2 of 344 patients diagnosed with RBD [15].
(2) Missense variations in the *glucosylceramidase beta* (*glucocerebrosidase* or *GBA)* gene (chromosome 1q22; gene ID 2629, MIM 606463), related with PD and DLBD, have been found in 7 of 69 iRBD patients (11.6%) and in 1 of 84 healthy matched controls (1.2%, *p* = 0.026) [16].
(3) A sequencing study of 25 genes previously identified in Genomic Wide Association Studies (GWAS) of PD involving 1039 iRBD patients and 1852 controls found an association of rare coding heterozygous nonsynonymous variants in the *bone marrow stromal cell antigen 1* (*BST1*; chromosome 4p15.32; gene ID 683, MIM 600387; implicated in facilitation of pre-B-cell growth) and rare noncoding variants in the *lysosomal associated membrane protein 3* (*LAMP3*) genes (chromosome 3q27.1; gene ID 27074; MIM 605883; implicated in the induction of primary T-cell response) [17].
(4) A study involving 347 RBD patients and 347 matched controls showed that, compared with controls, RBD patients were more likely to smoke, to report a previous head injury, and to have worked as farmers, with a borderline increase in welding, to have previously occupational exposure to pesticides, and to have few years of formal schooling, while there were no significant differences in coffee consumption [18].

This narrative review focuses in provide an extensive description of studies published related with the neurochemistry and biochemical findings of RBD.

## 2. Search Strategy

The references used for this review were identified through a PubMed search which including the period from 1966 until 31 July 2021. The term “*REM sleep behavior disorder*” was crossed with “*neurochemistry*” (5 items), “*biochemistry*” (22 items), “*neurotransmitters*”(321 items), “*dopamine*” (326 items), “*noradrenaline*” (32 items), “*norepinephrine*” (32 items), “*serotonin*” (55 items), “*acetylcholine*” (22 items), “*GABA*”(54 items), “*gamma-aminobutyric acid*”(18 items), “*histamine*” (2 items), “*glycine*”(15 items), “*glutamate*” (17 items), “*neuropeptides*”(39 items), “*white matter*”(14 items), “*gray matter*”(23 items), “*cortical thickness*”(10 items), “*neuroimaging*” (176 items), and “*magnetic resonance neuroimaging*” (66 items). We made a selection (after examining one-for-one) of the references strictly related to the neurochemical findings in RBD (a total of 180) from the 537 references retrieved by the whole search.

### 2.1. Dopaminergic Dysfunction

Table 1 summarizes the results of functional neuroimaging studies with Single Photon Emission Computerized Tomography (SPECT) or Positron Emission Tomography (PET) reported to date in patients diagnosed with RBD, including those using different methods and tracers for the presynaptic dopaminergic (DA) terminal and postsynaptic dopamine D_2_ receptors (DRD_2_). The majority of studies analyzing the presynaptic DA terminal have shown a significant decrease in the striatal tracer uptake in many patients diagnosed, at least initially, with iRBD [19,20,21,22,23,24,25,26,27,28,29,30,31,32,33,34,35,36,37,38,39,40,41,42,43,44], with some exceptions [45], being this decrease lower than that found in patients diagnosed with PD [27,28,31,43,45] or LBD [43] with or without concomitant RBD, although PD patients with versus those without RBD did not show significant differences [46]. However, patients with and without decreased striatal tracer uptake did not differ in clinical features according to one study [32], while in others, iRBD patients with mild motor impairment or with higher Unified Parkinson’s Disease Rating Scale (UPDRS) motor score showed decreased striatal tracer uptake when compared with those without mild motor impairment and with controls [33,34]. Patients with iRBD and MCI showed more frequently decreased striatal tracer uptake [37]. Follow-up studies have shown that many patients initially diagnosed with iRBD and reduced striatal tracer uptake at the presynaptic DA terminal at baseline developed neurodegenerative disorders such as PD, DLB, and MSA [21,22,30,40]. In contrast, studies on postsynaptic DRD_2_ have not shown significant differences in the tracer uptake in patients with iRBD compared with controls [38,41]. A meta-analysis of studies published up to 2018 on the presynaptic dopaminergic terminal neuroimaging in RBD patients showed that tracer uptake progressively decreased from controls to iRBD and eventually PD patients with RBD at the putamen level, while tracer uptake at caudate overlapped between patients with iRBD and those with PD without RBD [47].

Substantia nigra (SN) hyperechogenicity (SNH), assessed by transcranial sonography (TCS), a finding that likely reflects increased iron content in the SN, is considered as a useful diagnostic marker for nigrostriatal degeneration in PD. Because an important percentage of patients initially diagnosed with iRBD develop PD or other synucleopathies, this technique should be a useful tool to predict conversion of iRBD to PD and other neurodegenerative diseases. SNH was significantly more frequent in patients diagnosed with iRBD than in controls [21,48,49,50], although less frequent than in patients diagnosed with PD [48,51], and had a similar frequency than that reported in MSA patients [51]. SNH was more frequent in iRBD patients with than in those without mild motor abnormalities [24]. Iranzo et al. [21] found SNH in 36% of 39 patients with iRBD and in 11% of 149 controls, and described a sensitivity of 100% and a specificity of 55% of the combined use of TCS with ^18^F-N-(3-fluoropropyl)-2beta-carbon ethoxy-3beta-(4-iodophenyl) nortropane (^123^I-FP-CIT) SPECT for the prediction of conversion of iRBD to synucleopathies after 2–5 years of follow-up, while the use of TCS alone had a sensitivity of 42.1% and a specificity of 67.7% to make this prediction after 5 years of follow-up [52]. Miyamoto et al. [53] described a higher phenoconversion rate to other synucleopathies in patients with SNH (57.4%) than in those with normal TCS (25.0%). Patients with SNH showed decreased 6-^18^F-fluoro-metatyrosine (FMT) striatal uptake compared with those with normal TCS in another study [44]. In addition, an important percentage of iRBD patients (50%) showed basal ganglia hyperechogenicity, a finding that was less frequent in PD patients (18.2%) and more frequent in MSA patients (66.7%) [51]. SNH, combined with hypoechogenicity of the brainstem raphe, has been described as useful to detect comorbid depression in iRBD patients [54].

It has been described an anecdotal report of RBD induced by quetiapine [55]. This drug acts as DRD_1_ and DRD_2_ antagonist, but also as serotonin (5-hydroxytryptamine—5-HT) 1 and 2 (5-HT_1_ and 5-HT_2_), histamine H_1_, and α1 and α2-adrenergic receptors antagonist. Therefore, it is unknown if the ability of this drug to induce RBD could be related to the dopaminergic system.

Rats with hemiparkinsonism induced by unilateral lesions of the nigrostriatal system with 6-hydroxydopamine (6-OH-DA) showed when compared with normal rats, more REM epochs with muscle activity similar to that found in PD patients with sleep disorders including RBD, suggesting a role of dopaminergic deficit in the pathogenesis of RBD in this model [56]. This is also suggested by the description of improvement of RBD symptoms in PD patients under therapy with levodopa [5,57] or with the monoamine oxidase B (MAOB) inhibitor selegiline [58], and the improvement of iRBD with the dopamine agonist pramipexole [59,60,61,62].

**Table 1:** Results of functional neuroimaging studies using dopamine, noradrenalin, serotonin, acetylcholine, inflammatory tracers, and amyloid in patients with REM sleep behaviour disorder RBD. ^11^C-CFT: 2b-carbomethoxy-3b-(4-trimethylstannylphenyl) tropane: 11[C]DTBZ [^11^C]dihydro-tetrabenazine; ^11^C-PK11195 (1-[2-chlorophenyl]-N-[1-methyl-propyl]-3-iso-quinoline; carboxamide); DASB ^11^C3-Amino-4-(2-dimethyl-aminome-thyl-phenylsulfa-ryl)-benzonitrile; DAT dopamine transporter; DNH dorsal nigral hyperintensity; EMG electromyography; ^18^F-AV133 9-^18^F-fluoropropyl-(+)-dihydrotetra-benazine; ^18^F-FP-CIT: ^18^F-N-(3-fluoropropyl)-2beta-carbon ethoxy-3beta-(4-iodophenyl) nortropane; F-DOPA: ^18^Fluoro-L-Dopa; FEOBV ^18^F-fluoroethoxy-benzovesamicol; FMT 6-[(^18^)F] fluoro-metatyrosine; ^123^I-beta-CIT: ^123^I -2β-carbomethoxy-3β-(4-iodophenyl)-nortropane (^123^I-ioflupane); IBZM: (S)-2-hydroxy-3-iodo-6-methoxy-[(1-ethyl-2-pyrrolidinyl) methyl] benzamide; ^123^I-IBVM ^123^I-iodobenzove-samicol; IPT: N-(3-iodopropen-2-yl)-2beta-carbomethoxy-3beta-(chloro-phenyl) tropane; iRBD idiopathic RBD; MCI mild cognitive impairment; MDS Movement Disorders Society; MeNER ^11^C-methyl-reboxetine; MoCA Montreal Cognitive Assessment; MRI Magnetic Resonance Imaging; MSA multisystem atrophy; PD Parkinson’s disease; PET: Positron Emission Tomography SPECT: Single Photon Emission Computerized Tomography; RBD: REM sleep behaviour disorder: SN substantia nigra; TCS transcranial stimulation; ^18^F-TRODAT-1Technetium-99m, [2-[[2-[[[3-(4-chlorophenyl)-8-methyl-8-azabicyclo[3.2.1]oct-2-yl]methyl] (2-mercaptoethyl)amino]eth-yl]amino]ethanethiolato(3-)-N2,N2′,S2,S2′]oxo-[lR-(exo-exo)]; UPDRS Unified PD Rating Scale; UPSIT University of Pennsylvania Smell Identification Test: WASO wake after sleep onset.

	Method	Authors, Year [Ref]	RBD/ Controls	Main Findings
**Presynaptic DA terminal**	^123^I-beta-CIT-SPECT	Stiasny-Kolster et al., 2005 [63]	30 (6 iRBD, 13 symptomatic, 11 subclinical)/30	The main objective of the study was the demonstration of olfactory dysfunction. 11 patients underwent SPECT studies Decreased uptake of the tracer in the striatum in 1 patient with PD + RBD and 2 with iRBD from 11 patients who underwent SPECT study,
	^123^I-beta-CIT-SPECT	Unger et al., 2008 [19]	5/0	Decreased uptake of the tracer in the striatum in the 5 RBD patients (2 patients showed SN hyperechogenicity in the right side)
	^123^I-beta-CIT-SPECT	Miyamoto et al., 2010 [64]	1/6	Decreased uptake of the tracer in the striatum in the RBD patients. Decreased rate of the uptake of the tracer by 4–6% annually during 2.5 years of follow-up in the RBD patient.
	^123^I-beta-CIT-SPECT	Kim et al. [20]	14/12 (+14 PD patients)	Decreased tracer striatal uptake (specially in the putamen) in RBD patients. Normal DAT densities in the putamen of 11 RBD patients. Lack of correlation between EMG activities and DAT densities in RBD patients
	^123^I-beta-CIT-SPECT	Iranzo et al., 2010 [21]	43/18 (149 for transcranial sonogra-phy—TCS-studies)	Reduced binding of the tracer in the striatum by 40% of iRBD patients compared with controls, which was more marked in the putamen than in the caudate nucleus. SN hyperechogenicity in 14/39 (36%) of 39 iRBD patients and in 16/149 (11%) controls. 63% iRDB patients had reduced ^123^I-FP-CIT binding or SN hyperechogenicity at baseline. While individuals with normal neuroimaging results remained disease-free, eight iRDB patients developed PF (5), LBD (2), or MSA (1)-. The combined use of ^123^I-FP-CIT SPECT and TCS was able to predict the conversion to a synucleinopathy 2–5 years later with a sensitivity of 100% and a specificity of 55%.
	^123^I-beta-CIT-SPECT	Iranzo et al., 2011 [22]	20/20 (study at baseline and after 1.5 and 3 years)	iRBD patients had significantly reduced mean tracer binding in all four striatal regions at baseline (in 10 patients) and after 3 years (in 13 patients). The mean reduction in tracer uptake from baseline to 3 years was 19·36% in the left putamen, 15·57% in the right putamen, 10·81% in the left caudate nucleus, and 7·14% in the right caudate nucleus. The decline in tracer binding at baseline to 3 years was significantly greater in patients than in controls in all studied regions except for the right caudate nucleus. At the 3-year assessment, three patients were diagnosed with PD (these patients had the lowest tracer uptake at baseline)
	^123^I-beta-CIT-SPECT	Mossa et al., 2012 [23]	5/5	All RBD patients showed reduced tracer striatal binding.
	^123^I-beta-CIT-SPECT	Rupprecht et al., 2013 [24]	28/0 (18 iRBD patients showed mild motor abnormalities)	The main objectives of the study were the demonstration of mild motor abnormalities, olfactory dysfunction, and SN hyperechogenicity by TCS. 11 patients underwent SPECT studies. 15 patients showed SN hyperechogenicity Decreased uptake of the tracer in 4 of 11 patients (this was not associated to SN hyperechogenicity, and with olfactory dysfunction, but subjects with decreased tracer uptake showed higher scores in a motor symptoms scale)
	^123^I-beta-CIT-SPECT	Arnaldi et al., 2015 [25]	23/23	Decreased uptake of the tracer in the basal ganglia.
	^123^I-beta-CIT-SPECT	Arnaldi et al., 2015 [26]	12/0 (+16 patients with PD and 24 with PD + RBD)	iRBD patients showed higher putamen-specific binding ratio values than PD patients with and without RBD, whereas the difference between PD groups was not significant. PD with RBD patients showed higher caudate-specific binding ratio than patients with PD without RBD and iRBD patients.
	^123^I-beta-CIT-SPECT	Zoetmulder et al., 2016 [27]	10/10 (+10 patients with PD and 10 with PD + RBD)	Uptake of the tracer was highest in controls, followed by iRBD patients, and lowest in PD patients. When compared to controls both iRBD and PD patients with RBD showed increased EMG-activity. In iRBD patients EMG-activity in the mentalis muscle was correlated with tracer uptake.
	^123^I-beta-CIT-SPECT	Rolinski et al., 2017 [28]	26/23 (+48 PD patients)	The main aim of the study was to assess basal ganglia connectivity. Eight RBD patients, 10 PD patients and 10 controls underwent SPECT. Compared with controls PD patients showed reduced tracer uptake in the 5 regions of interest (striatum, caudate, putamen, anterior and posterior putamen relative to occipital cortex), while RBD patients showed a trend towards reduced uptake. PD patients showed reduced tracer uptake compared with RBD patients in the striatum, caudate, putamen, and posterior putamen
	^123^I-beta-CIT-SPECT	Meles et al., 2017 [29]	21/19 (+20 patients with PD and 22 with DLB)	The main objective of the study was the study of brain glucose metabolism 9 of 21 iRBD patients showed loss of striatal DAT binding in the putamen.
	^123^I-beta-CIT-SPECT	Frosini et al., 2015 [65]	15/14 (+28 PD patients)	Decreased uptake of the tracer in the striatum in 9 RBD patients (8 of them showed abnormalities on 7T-MRI).
	^123^I-beta-CIT-SPECT	Iranzo et al., 2017 [30]	20/20 (follow-up during 5.7 ± 2.2 years)	58.5% iRBD patients showed baseline DAT deficit 25 (28.7%) subjects developed clinically defined synucleinopathy (11 PD, 13 LBD, 1 MSA) during the follow-up period, with a mean latency of 3.2 ± 1.9 years from imaging. Subjects with abnormal baseline DAT-SPECT showed an increased risk of incident synucleinopathy during the follow-up period (reduced uptake greater than 25% in putamen discriminate patients at this risk).
	^123^I-beta-CIT-SPECT	Bae et al., 2018 [31]	18/18 (+18 PD patients)	Decreased tracer uptake ratios in the 11 patients with iRBD and nigral hyperintensity loss in 3.0 Tesla-MRI NH compared with control, but higher tracer uptake than PD patients. Five patients with iRBD and nigral hyperintensity loss developed parkinsonism or dementia 18 months after neuroimaging
	^123^I-beta-CIT-SPECT	Barber et al., 2018 [66]	43 (18 of them with apathy)	Lack of correlation between the severity of apathy and the tracer uptake in the basal ganglia.
	^123^I-beta-CIT-SPECT	Chahine et al., 2019 [32]	75/0	46 patients with and 29 patients without decreased tracer did not differ in clinical features (age, gender, education, right vs left-handedness, mean disease duration, presence of REM sleep without atonia, UPDRS scores, and MoCA scores)
	^123^I-beta-CIT-SPECT	Yamada et al., 2019 [33]	23 (8 with mild motor impairment)/20	Decreased tracer uptake in the right posterior putamen, bilateral anterior putamen, and caudate in iRBD patients with mild motor impairment (in finger tapping) when compared with iRBD patients with normal motor function or with controls.
	^123^I-beta-CIT-SPECT	Dušek et al., 2019 [34]	74/39	The main objective of the study was the assessment of motor impairment (MDS-UPDRS), cognitive impairment (MoCA), olfactory dysfunction (UPSIT), autonomic impairment, depression, and anxiety. 65 patients underwent DAT-SPECT. RBD with abnormal DAT-SPECT had a higher MDS-UPDRS motor score and higher prevalence of orthostatic hypotension Putaminal binding ratio positively and negatively associated, respectively, with UPSIT score and tonic and phasic muscle activity during REM sleep.
	^123^I-beta-CIT-SPECT	Barber et al., 2020 [35]	46/32 (+28 PD patients)	The main objective of the study was the assessment of dorsal nigral hyperintensity (DNH) using susceptibility-weighted MRI. 42 RBD patients underwent SPECT study (14 of 36 -39%- patients with DNH had reduced tracer uptake)
	^123^I-beta-CIT-SPECT	Li et al., 2020 [36]	15/7	Decreased uptake of the tracer in the bilateral putamen and left caudate striatum in iRBD patients compared with controls.
	^123^I-beta-CIT-SPECT	Arnaldi et al., 2021 [67]	263/243	Fifty-two (20%) iRBD patients developed a synucleinopathy after an average follow-up of 2 years. The presence of putamen dopaminergic dysfunction of the most affected hemisphere on imaging, together with age over 70 years, and constipation, was the best combination of risk factors to precict phenoconversion. Development of parkinsonism was more frequent in iRBD patients with lower caudate binding asymmetry and higher Mini-Mental State Examination scores, while development of dementia was more likely in patients with the opposite pattern.
	^123^I-beta-CIT-SPECT	Mattiolli et al., 2021 [37]	39 (17 with mild cognitive impairment)	The main objective of the study was the assessment of cerebral glucose metabolism and brain connectivity. 48.7% iRBD patients showed decreased tracer uptake in caudate and/or putamen (82% of those with vs 23% of those without MCI)
	^123^I-IPT-SPECT	Eisensehr et al., 2003 [38]	16 (8 subclinical)/11 (+8 patients with early PD)	Decrease in 123IPT striatal uptake in subclinical RBD when compared with controls, in clinical RBD compared with subclinical RBD, and PD patients compared with patients with clinical RBD. Non-significant differences in IBZM striatal uptake among groups.
	^99m^TC-TRODAT-1 SPECT	Rizzo 2018 [39]	6/0	5 of 6 iRBD patients showed decreased tracer striatal uptake
	^18^F-FP-CIT PET	Yoon et al., 2019 [45]	28/24 (+21 PD patients with RBD)	Non-significant decrease in tracer striatal uptake in iRBD patients compared with controls. PD + RBD patients showed significant decreased striatal tracer uptake compared with iRBD patients and with controls
	^18^F-FP-CIT PET	Shin et al., 2020 [40]	39/19 (+31 drug-naïve PD patients)	Baseline decreased tracer striatal uptake in iRBD patients when compared with controls, and in PD patients compared with iRBD patients and with controls. After a follow-up period of 5 years 10 iRBD patients converted to neurodegenerative disease (5 PD, 1 MSA, and 4 LBD), and 6 dropped-out from the study. The baseline PD pattern of DAT predicted 58% of disease converters (67% if combined with the presence of hyposmia).
	^18^Fluoro-L-Dopa (F-DOPA) PET	Wing et al., 2015 [41]	11 (with comorbid major depressive disorder)/10 (+8 with comorbid major depressive disorder without RBD)	Patients with RBD and comorbid major depressive disorder had decreased tracer uptake in comparison with controls and with patients with major depressive disorder alone.
	^18^Fluoro-L-Dopa (F-DOPA) PET	Stokholm et al., 2018 [42]	21/9	Decreased tracer uptake in the thalamus of iRBD patients compared with controls. Non-significant differences in tracer uptake between iRBD and control groups in other extrastriatal regions.
	^18^Fluoro-L-Dopa (F-DOPA) PET	Gersel Stokholm et al., 2018 [68]	17/9	Decreased tracer uptake in the caudate and putamen of iRBD patients compared with controls.
	^18^Fluoro-L-Dopa (F-DOPA) PET	Knudsen et al., 2018 [69]	17/14 (+8 PD patients)	Decreased tracer uptake in 21% of iRBD patients and a significant decreased tracer uptake in PD patients compared with iRBD and with controls
	[^11^C]dihydro-tetrabenazine ([^11^C]DTBZ) PET	Gillman et al., 2003 [70]	13 (RBD + MSA)/15	Decreased tracer binding in the striatum in patients with RBD associated with MSA. In subjects with RBD + MSA the severity of REM atonia loss was inversely correlated with the striatal tracer uptake.
	[^11^C]dihydro-tetrabenazine ([^11^C]DTBZ) PET	Kotagal et al., 2012 [46]	80 PD patients (27 of them with RBD)	Twenty-seven of 80 subjects (33.8%) indicated a history of RBD symptoms. Non-significant differences between PD patients with and without RBD in striatal tracer binding.
	9-^18^F-fluoropropyl-(+)-dihydrotetra-benazine (^18^F-AV133) (assessing VMAT2)	Beauchamp et al., 2020 [43]	14/16 (+20 PD + 10 DLB patients)	Decreased striatal tracer uptake in RBD patients compared with controls, and even higher decreased tracer uptake in PD and LBD patients
	6-[(18)F] fluoro-metatyrosine (FMT) PET	Miyamoto et al., 2012 [44]	19 (9 with SN hyperechogenicity by TCS)	Decreased striatal tracer uptake in iRBD patients than in those without SN hyperechogenicity compared with those without SN hyperechogenicity. SN echogenicity and tracer uptake were not correlated.
	6-[(18)F] fluoro-metatyrosine (FMT) PET	Miyamoto et al., 2020 [71]	24 (follow-up during 1–10 years	11 iRBD patients (45.8%) developed PD (6) or LBD (5). Compared to iRBD who were still disease-free, those who developed PD or LBD with parkinsonism showed significantly reduced bilateral putaminal FMT uptake during the follow-up. iRBD patients who converted to PD or DLB showed a higher rate of FMT decline between baseline and follow-up scans.
	^11^C-CFT PET	Huang et al., 2020 [72]	37/15 (+86 PD patients)	The main objective of the study was the assessment of cerebral glucose metabolism. Decreased striatal tracer uptake in iRBD and PD patients compared with controls (iRBD patients presented with an intermediate state in striatal DAT between PD and normal controls).
**Postsynaptic DA terminal**	^123^I-IBZM-SPECT (striatal D_2_ receptors)	Eisensehr et al., 2003 [38]	16 (8 subclinical)/11 (+8 patients with early PD)	No significant differences in tracer uptake among the 3 study groups.
	^11^C-raclopride PET (striatal D_2_ receptors)	Wing et al., 2015 [41]	11 (with comorbid major depressive disorder)/10 (+8 with major depressive disorder without RBD)	No significant differences in the striatal tracer uptake among the 3 study groups.
**Noradrenalin transporter**	^11^C-methyl-reboxetine (MeNER) PET	Knudsen et al., 2018 [69]	14/9 (+22 PD patients)	Significant reduction of in the left thalamic tracer uptake in iRBD patients compared with controls but similar uptake than patients with PD Tracer uptake of the red nucleus in iRBD patients similar to that of controls and PD patients.
	^11^C-methyl-reboxetine (MeNER) PET	Andersen et al., 2020 [73]	17/25 (+30 PD patients, 16 of them with RBD)	Reduced tracer uptake in the primary sensorimotor cortex in the 3 study groups compared with controls. Significant correlation between putaminal 18F-DOPA uptake and 11C-MeNER uptake in iRBD patients.
**Serotonin transporter (SERT)**	^123^I-beta-CIT-SPECT	Arnaldi et al., 2015 [25]	23/23	No significant differences in tracer uptake between iRBD and normal subjects at brainstem and thalamus levels, suggesting that the serotoninergic system is not involved in iRBD.
	^123^I-beta-CIT-SPECT	Barber et al., 2018 [66]	43 (18 of them with apathy)	Correlation between the severity of apathy in RBD patients and the tracer uptake in the dorsal raphe nuclei.
	^11^C3-Amino-4-(2-dimethyl-aminome-thyl-phenylsulfa-ryl)-benzonitrile (DASB) PET	Kotagal et al., 2012 [46]	80 PD patients (27 of them with RBD)	Twenty-seven of 80 subjects (33.8%) indicated a history of RBD symptoms. Non-significant differences between PD patients with and without RBD in the brainstem and striatal tracer binding.
**Acetylcholine transporter**	^123^I-iodobenzove-samicol (^123^I-IBVM) PET	Gillman et al., 2003 [70]	13 (RBD + MAS)/12	Patients with RBD + MSA showd decreased tracer binding in the thalamus
	^18^F-fluoroethoxy-benzovesamicol (FEOBV) PET	Bedard et al., 2019 [74]	5/5	Increased FEOBV uptake in patients with iRBD, specially in specific brainstem areas corresponding to the mesopontine cholinergic nuclei, tegmental periacqueductal gray pontine coeruleus/subcoeruleus complex, and, bulbar reticular formation, but also in some cortical territories (orbitofrontal and anterior cingulate cortex, and the paracentral lobule), deep cerebellar nuclei and the ventromedial area of the thalamus. Significant correlation between muscle activity during REM sleep and increased uptake in the mesopontine area and paracentral cortex.
**Postsynaptic acetylcholine terminal**	^11^Cmethylpiperidylpropionate acetyl-cholinesterase PET	Kotagal et al., 2012 [46]	80 PD patients (27 of them with RBD)	Twenty-seven of 80 subjects (33.8%) indicated a history of RBD symptoms. PD patients with RBD, in comparison to those without, exhibited decreased neocortical, limbic cortical, and thalamic cholinergic innervation
	^11^C-donepezil PET	Gersel Stokholm et al., 2020 [68]	17/9	Compared with controls, patients with iRBD showed a mean 7.65% reduction in neocortical tracer. The most significant reductions were found at the bilateral superior temporal cortex, occipital cortex, cingulated cortex, and dorsolateral prefrontal cortex.
	^11^C-donepezil PET	Staer et al., 2020 [75]	19/27	Significant reduction of the tracer uptake in a global cortical region and a trend toward reduction in the substantia innominata in iRBD patients. Negative correlation between a lower cortical 11C-donepezil uptake (specially in the frontal and temporal lobes) and a higher 11C(R)-PK11195 binding in the substantia innominata. Lack of correlation between tracer uptake and iRBD duration.
**Inflammation markers (microglia activation)**	^11^C-PK11195 positron emission tomography (PET)	Stokholm et al., 2018 [42]	21/20	No significant differences in tracer uptake between iRBD and controls in the thalamus. Decreased tracer uptake in the occipital region in iRBD patients.
	^11^C-PK11195 positron emission tomography (PET)	Staer et al., 2020 [75]	19/27	No significant differences in tracer uptake between iRBD and controls in the substantia innominata in iRBD patients. Negative correlation between a lower cortical 11C-Donepezil uptake (specially in the frontal and temporal lobes) and a higher 11C(R)-PK11195 binding in the substantia innominata. Lack of correlation between tracer uptake and iRBD duration.
**Amyloid**	18F-flutemetamol amyloid PET	Lee et al., 2020 [76]	23 (4 with and 19 without amyloid deposits)	Patients with amyloid deposits showed a higher percentage of stage N1 and stage N2 sleep and of WASO, and scored worse on the Stroop Word Color Test compared to amyloid negative patients. Global tracer uptake was correlated with total sleep time, sleep efficiency, WASO, and N1 sleep (these sleep parameters were associated with a part of the default mode network of brains such as left temporal. dorsolateral pre-frontal, and orbitofrontal areas).

### 2.2. Noradrenaline

The possible role of noradrenaline in RBD could be suggested by the induction of this disorder by noradrenaline reuptake inhibitors such as mirtazapine [77] or duloxetine [78] (however, these drugs act as mixed serotonin-noradrenaline reuptake inhibitors), and by the beta-blocker bisoprolol [79].

*Post-mortem* studies have shown a severe monoaminergic cell loss in the *locus ceruleus* (noradrenergic) of a patient with RBD [80]. Another study did not find a significant reduction of tyrosine-hydroxylase (precursor of DA and NA) in the *locus ceruleus* of patients with LBD with or without concomitant RBD [81]. Schenk et al. [82] did not identify reactivity with *locus ceruleus* or any other brainstem area with sera from RBD patients, suggesting a lack of association of human RBD to anti-*locus ceruleus* antibodies.

Table 1 summarizes the results of PET studies using noradrenaline transporter ligands in iRBD patients. One of them showed reduced binding of this tracer in the primary sensorimotor cortex in iRBD patients and in PD patients with and without RBD compared with controls, and a significant correlation between putaminal ^18^F-fluorodihydroxyphenyl alanine (^18^F-DOPA) uptake and ^11^C-methyl-reboxetine (^11^C-MeNER) uptake in iRBD patients [73]. Another PET study with this tracer showed a significant reduction of in the left thalamic ^11^C-MeNER uptake in iIRBD patients compared with controls but similar uptake than patients with PD, while ^11^C-MeNER uptake of the red nucleus was similar to that of controls and PD patients [69].

Many studies have shown decreased myocardial ^123^I-metaiodobenzylguanidine (^123^I-MIBG) uptake (assessed by the hearth/mediastinum ratio) in patients diagnosed with iRBD compared with controls, suggesting loss of sympathetic noradrenergic terminals [69,83,84,85,86,87,88,89,90,91,92,93,94,95]. This finding is similar to that previously described in PD and DLB (but not in MSA and PSP). Patients with iRBD showed even more ^123^I-MIBG decreased uptake than patients with early PD [91]. Exceptionally, it has been described ^123^I-MIBG decreased uptake in patients diagnosed with iRBD with normal dopamine transporter (DAT) SPECT scan with ^123^I-FP-CIT [89,90]. Patients with RBD associated with narcolepsy have not shown decreased ^123^I-MIBG uptake, therefore suggesting that narcolepsy-RBD and iRBD have different pathophysiology [94].

Finally, a recent study has shown lower supine norepinephrine plasma levels in iRBD patients compared with controls [96].

### 2.3. Serotonin

The induction of RBD by tricyclic antidepressants [97,98], selective serotonin reuptake inhibitors [97,98,99,100,101,102], or by mixed serotonin-noradrenalin reuptake inhibitors [77,78], suggests a possible role of serotonin in the pathophysiology of this disorder. Antidepressant therapy has been associated with elevated REM sleep without atonia, both in patients with and without RBD [103]. Moreover, 12.2% of subjects of a cohort of 1444 subjects under antidepressant therapy showed REM sleep without atonia in PSG studies, a significantly higher frequency than that found in 10746 subjects undergoing PSG in a sleep laboratory (2.1%; OR [95%] CI = 9.978 [8.149–12.22]) [104]. Some authors suggested that the development of RBD with antidepressant therapy should be an early signal of underlying neurodegenerative disease [102]. In contrast, an anecdotal report described improvement of RBD with selective serotonin reuptake inhibitors and worsening with the 5-HT_1A_ partial agonist in a single patient [105].

Table 1 summarizes the results of PET studies using serotonin transporter (SERT) ligands in iRBD patients. A study using ^123^I-FP-CIT-SPECT as a marker of both DAT and SERT at the basal ganglia and of at brainstem and thalamus showed non-significant differences between iRBD patients and controls at brainstem and thalamus levels, suggesting the lack of direct implication of the serotonergic system in RBD [25]. Another study showed a correlation between the severity of apathy in RBD patients and the tracer uptake in the dorsal raphe nuclei [66]. Finally, a study using ^11^C3-Amino-4-(2-dimethyl-aminome-thyl-phenylsulfaryl)-benzonitrile (DASB) PET, showed non-significant differences between PD patients with and without RBD in the brainstem and striatal tracer binding [46].

### 2.4. Acetylcholine

It has been reported induction of RBD by the acetyl-cholinesterase inhibitor rivastigmine in a patient diagnosed with Alzheimer’s disease [106], while a double-blind, crossover trial involving 12 patients with PD and RBD found improvement of RBD with this drug [107], therefore suggesting a possible role of the cholinergic system in this effect. A neuropathological study showed a marked reduction in choline-acetyl-transferase labeling of neurons of the pedunculopontine/laterodorsal tegmentum nucleus in patients diagnosed with LBD, which was similar for those with or without concomitant RBD [81].

The results of brain PET studies using tracers for acetylcholine transporter and the postsynaptic acetylcholine terminal are summarized in Table 1. Patients with iRBD have shown decreased uptake of acetylcholine transporter, mainly but not exclusively, in several brainstem areas (reticular formation, pontine coeruleus/subcoeruleus complex, tegmental periaqueductal grey, and mesopontine cholinergic nuclei), which was correlated with muscle activity during REM sleep [74]. Patients with RBD associated with MSA have shown decreased acetylcholine transporter binding in the thalamus [74]. Studies on the postsynaptic acetylcholine terminal have shown reduced tracer uptake in several cortical areas in iRBD patients [68,75], and decreased neocortical, limbic cortical, and thalamic tracer uptake in PD patients with RBD compared to those without RBD [46]. Patients with RBD have decreased colonic uptake of ^11^C-donepezil, suggesting loss of cholinergic gut innervation [69].

The first study with proton magnetic resonance spectroscopy (^1^H-MRS) showed an increased choline/creatine ratio (choline is the precursor of acetylcholine) in a patient with RBD [108]. A further study involving 15 iRBD patients and 15 controls did not find significant differences between these two groups [109]. Similarly, non-significant differences were found comparing 12 patients with PD and RBD with 12 patients with PD without RBD [110]. However, a recent study involving 18 iRBD patients, 26 secondary RBD patients, and 29 controls, found a significant reduction in the same ratio in both groups of patients diagnosed with RBD [111].

Studies on the short-latency afferent inhibition (SAI) of the motor cortex with transcranial magnetic stimulation (which gives direct information about the function of some cholinergic circuits in the brain) has shown a mean reduction of this value in iRBD patients compared with controls, and a correlation between SAI and tests measuring episodic verbal memory and executive functions [112]. SAI has also been found decreased in PD patients suffering from RBD as compared both, with PD patients without RBD and with control subjects [113].

Finally, it has been described, in transgenic mice modeling brain amyloid pathologies, a significant reduction in pedunculopontine tegmentum choline-acetyl-transferase positive neurons related with impaired REM sleep [114].

### 2.5. Other Neurotransmitter Systems, Neuropeptides, and Hormones

#### 2.5.1. Aspartate, Glutamate, Gamma-Amino-Hydroxybutyric Acid (GABA), and Glycine

Two studies using ^1^H-MRS have shown non-significant differences of N-acetyl-aspartate (NAA)/creatinine ratio in the midbrain and in the pontine tegmentum between iRBD patients and controls [109,110]. Another group found a significant decrease in NAA/Creatinine ratio in secondary RBD patients compared with iRBD and controls, and significant decrease in NAA/Choline in secondary RBD and iRBD patients compared with controls [111].

Clément et al. [115] showed in rats that the neurons of the sublaterodorsal tegmentum which triggers paradoxical REM sleep are glutamatergic and are implicated in REM sleep atonia during this sleep stage through their descending projections to medullary and spinal glycinergic premotor neurons.

The injection of adeno-associated viral vectors modifying *vesicular GABA-glycine transporter* (*VGAT)* or *vesicular glutamate transporter 2* (*VGLUT2*) genes in mice have shown the contribution of glycinergic/GABAergic interneurons of the spinal ventral horn and glutamatergic neurons of the sublaterodorsal nucleus to REM atonia and of a separate population of glutamatergic neurons in the caudal laterodorsal tegmental and sublaterodorsal nuclei to REM sleep generation, while presynaptic GABA release in the caudal laterodorsal tegmental/sublaterodorsal nuclei, ventrolateral periaqueductal gray matter, and lateral pontine tegmentum were not involved in REM sleep control [116]. Genetic inactivation of glutamatergic neurons in the sublaterodorsal tegmentum nucleus of rats using adeno-associated viruses modifying VGLUT2 induces symptoms and behaviours during paradoxical sleep resembling human RBD [117].

Transgenic mice with deficient glycine and GABA neurotransmission show sleep, motor, and behavioral phenotype resembling clinical features of human RBD [118].

Local microinjections of glutamate in the SN of rodents bilaterally increases REM sleep, being this effect decreased by haloperidol (DA antagonist) and increased by bicuculline (GABA receptor antagonist) [119].

Injections of GABA_B_ receptor agonists into the inferior collicollus in rats increased phasic motor activity in slow-wave sleep and tonic muscle activity in REM sleep, a finding that was associated with clinical RBD-like activity, while GABA_B_ receptor antagonists did not induce changes [120].

#### 2.5.2. Adenosine

The description of exacerbation of RBD in a patient by chocolate ingestion suggested a possible role of adenosine in this disorder [121]. It has been reported that bilateral microinjections of adenosine A_2_ receptor (A_2_AR) antagonists and A_2_AR agonists into the rat olfactory bulb, and similarly inhibition and inactivation of A_2_AR, increased and decreased REM sleep respectively, and the inhibition or activation of A2AR neurons increased and decrease, respectively, REM sleep [122].

#### 2.5.3. Peptides and Hormones

*Hypocretin* (*orexin-A*, a well-established marker of narcolepsy). It has been reported normality of cerebrospinal fluid (CSF) levels of this peptide in 5 patients diagnosed with iRBD [123]. However, in 63 patients diagnosed with concomitant narcolepsy and RBD, hypocretin deficiency was a predictor of symptoms of RBD [124].

Measurements of the fasting and postprandial serum levels of the orexigenic peptide *ghrelin* (implicated in promoting gastrointestinal motility and influencing higher brain functions) in 20 healthy controls, 39 (including 19 drug-naïve) PD patients and 11 iRBD patients have shown a less pronounced recuperation of the decrease of this peptide in the early postprandial phase in iRBD and PD patients than in controls, suggesting that ghrelin excretion should be a peripheral biomarker of both diseases [125].

A study assessing the postprandial secretion of *pancreatic polypeptide* and *motilin* involving 10 iRBD patients, 38 PD patients (19 of them drug-naïve), and 10 controls, showed a physiological pattern in all study groups, with a mild enhanced response in PD and iRBD [126].

A study assessing the 24-h blood *melatonin* profiles in 10 RBD patients and 10 controls showed delayed melatonin secretion by 2 h in the RBD group [127].

Finally, the serum levels of total, free, and bioavailable *testosterone* [128,129], and of *other hormones* including luteinizing hormone, follicle-stimulating hormone, estradiol-17 beta, sex-hormone binding globulin, and prolactin [129], are similar in iRBD patients than in controls.

### 2.6. Other Substances

#### 2.6.1. Uric Acid

Because higher plasma/serum uric acid levels have been associated with a lower risk for PD, several studies assessed plasma urate levels in patients with RBD. Plasma urate levels have been reported to be higher in patients with iRBD compared with those with PD with RBD, and a positive correlation between plasma urate levels and duration of iRBD has been found in a study [130]. However, another study found similar serum urate levels in iRBD patients and in controls, and described a longer duration of RBD and lower scores in attention, executive function, and language domains in patients with low urate levels [131].

A recent prospective study involving 12,923 Chinese adults showed an association between higher plasma urate levels and the risk for possible RBD [132].

#### 2.6.2. Proinflammatory Substances

The results of studies assessing the status of markers of microglia activation in RBD patients using 1-[2-chlorophenyl]-N-[1-methyl-propyl]-3-iso-quinoline; carboxamide (^11^C-PK11195) PET are summarized in Table 1. In summary, iRBD patients have shown decreased tracer uptake in the occipital region [68], similar tracer uptake than controls in the thalamus [68] and substantia innominata [75], and a negative correlation of higher ^11^C-PK11195 binding in the substantia innominata with lower cortical ^11^C-donepezil uptake, more marked in the frontal and temporal lobes [75].

Several studies assessed the serum or plasma levels of cytokines in patients with RBD. Kim et al. [133] described increased plasma levels of interleukin-10 (IL-10), and normal plasma levels of IL-1β, IL-2, IL-6, and tumor necrosis factor-α (TNF-α) in iRBD patients. Zhang et al. [134], described a significant increase of plasma TNF-α and IL-10 levels in iRBD, and decrease in plasma IL-6/IL-10 and IL-8/IL-10 ratios in iRBD patients compared with controls, and a higher predisposition to develop neurodegenerative synucleinopathies in iRBD patients with higher TNF-α/IL-10 after 3.7 years of follow-up. Finally, Kim et al. [135], found similar serum levels of IL-1β, IL-2, IL-6, IL-10, and TNF-α in iRBD patients and controls, but a higher risk of phenoconversion in patients with increased TNF-α levels and multiple markers.

A recent study showed increased blood classical monocytes and mature natural killer cells, a positive correlation of the levels of expression of Toll-like receptor 4 (TLR4) on blood monocytes in iRBD patients with nigral immune activation (assessed by ^11^C-PK11195 PET), and a negative correlation with putaminal ^18^F-DOPA uptake; and an opposite correlation with the percentage of Cluster of Differentiation 163 (CD163+) myeloid cells, suggesting a deleterious role for TLR4 and a protective one for the CD163 expression [136].

#### 2.6.3. Alpha-Synuclein

Determinations of α-synuclein as a possible marker for prodromal PD have been performed in several tissues of iRBD and PD patients and in controls. Immunostaining of α- synuclein was similar in the colonic mucosa and submucosa of iRBD patients and controls, but that of 129-phosphorylated-α-synuclein in submucosa nerve fibers or ganglia was found in 5.3% of 19 PD patients, 23.5% of 17 iRBD patients, and in 0% of controls [137].

Aggregates of 129-phosphorylated α-synuclein in the neurofibers or the parenchyma of the submandibular gland were detected in 89% of iRBD patients, 67% of PD patients, and in 0% of controls [138]; in 44.4% of RBD patients, 46.3% of PD patients and 10.2% of controls in olfactory mucose [139]; and in skin biopsies of 86.7% iRBD patients and in no patient with narcolepsy type 1 [140]. Another study showed positivity for α-synuclein in skin biopsies of 72% of iRBD patients [141].

Cerebrospinal fluid (CSF) α-synuclein assays by real-time quacking-induced conversion (Rt-QuIC) were positive in 90% of 52 iRBD patients and 10% of 40 controls, being diagnosed with PD 62% of iRBD patients after 7.1 years of follow-up [142].

Finally, hypomethylation of intron 1 of the *α-synuclein* (*SNCA*) gene was found more frequently in 78 iRBD patients than in 74 controls, being hypomethylation at cytosine-phosphate-guanine 17 associated with increased risk for clinical phenoconversion to neurodegenerative diseases and at cytosine-phosphate-guanine 14, 15, and 16 with disease progression [143].

#### 2.6.4. Lipoprotein and Protein Glycosylation Profile

Laguna et al. [144], using ^1^H NMR spectroscopy, assessed the lipoprotein profile and the presence of glycosylated proteins (a total of 27 metabolites) in the serum in 82 iRBD patients and 29 controls. Thirty-three iRBD patients showed iRBD only after 10.3 ± 4.1 years of follow-up, while 33 patients with apparent iRBD converted later to Lewy-type synucleinopathies, 35 had converted to Lewy-type synucleinopathies at the time of sample collection (20 to LBD and 15 to PD, a new sample were obtained after conversion). None of the metabolites measured differed significantly between iRBD patients who converted to Lewy-type synucleinopathies before and after this conversion and controls, but the subgroup of patients who converted to LBD showed decreased glycosylated protein B in comparison to control subjects. A comparison between samples of patients who converted to Lewy-type synucleinopathies obtained before and after this conversion showed higher levels of glycosylated protein B, and lower levels of medium sizes low-density lipoprotein (LDL) and of triglycerides in LDL after phenoconversion.

#### 2.6.5. Nasal and Gut Microbiome

A recent study comparing the nasal and gut microbiome of iRBD (*n* = 21) and PD patients (*n* = 76) with controls (*n* = 78), assessed by 16S and 18S ribosomal RNA amplicon sequencing, showed differentially abundant gut microbes in PD and in iRBD patients, while no strong differences were found in nasal microbiota. The presence of Anaerotruncus and several Bacteroides spp. correlated with nonmotor symptoms [145].

## 3. Brain Perfusion Studies

Table 2 summarizes the results of studies assessing cerebral blood flow and brain perfusion in patients with RBD by using different methods [146,147,148,149,150,151,152,153,154,155]. Most of them showed decreased cerebral blood flow in frontal and in temporoparietal cortex [146,148,149,152,154,155], and some of them increased cerebral blood flow in the pons [148,151], periaqueductal area [151], putamen [148], hippocampus [148], and cerebellum [151]. Others have shown decreased cerebral blood flow in the parieto-occipital lobe [147,151] and in the cerebellar hemispheres [147]. Several of the described patterns were similar to those described in PD and LBD [148,149,153].

According to several studies, alterations in brain perfusion were related to color discrimination tests [148,150], loss of olfactory discrimination [148], and global cognitive performance [149,155].

## 4. Brain Glucose Metabolism Studies

The results of studies assessing brain glucose metabolism in RBD patients, all of them by using ^18^F-fluoro-d-glucose positron emission tomography (^18^F-FDG), are summarized in Table 3 [29,37,41,45,72,92,156,157,158,159,160,161,162]. The majority of studies have shown diffuse areas of glucose hypometabolism, with a predominance in the occipital lobe (a pattern similar to that described in patients with LBD) [29,37,45,72,92,141,154,159,160,161,162].

The so-called PD-related pattern (PDRP), characterized by decreased cortical metabolic activity in the temporal, lateral occipital, inferior and posterior parietal, and prefrontal association cortices and in the supplementary motor area; and increased metabolism in the cerebellum and pons, pallidum, thalamus, paracentral lobule, limbic and sensorimotor cortices, and in the left supplementary motor area, was found with higher frequency in iRBD patients than in controls, but with lower frequency than in PD and LBD patients [29,159], while the iRBD-related pattern (iRBDRP, relative increased glucose metabolism in the brain stem, thalamus, cerebellum, hippocampus, and sensorimotor cortex, and decreased metabolism in the middle cingulated, parietal, temporal, and occipital cortices) was similar in iRBD and in PD patients and showed higher expression in both PD and iRBD patients than in controls [159]. A mixed PD and iRBD related pattern (PDRBDRP), which was more marked in PD patients than in iRBD patients and in both groups than in controls, has also been described [45]. In patients with iRBD and decreased DAT uptake and in patients with PD, but not in patients with iRBD and normal DAT uptake, DAT binding was correlated with PDRP expression [45]. The presence of *de novo* PDRBDRP (dnPDRBDRP) in iRBD patients seems to be a predictive marker for future phenoconversion [162].

It has also been described, both for iRBD and PD patients, increased glucose metabolism in the pons, thalamus, medial frontal and sensorimotor areas, hippocampus, supramarginal and inferior temporal gyri, and posterior cerebellum [156,157,161], and normal ^18^F-FDG uptake in the striatum of RBD patients [41].

## 5. Structural and Functional Magnetic Resonance Imaging (Mri) Studies

The results of studies assessing structural and functional MRI (fMRI) studies are summarized, in chronological order, in Table 4 [163,164,165,166,167,168,169,170,171,172,173,174,175,176,177,178,179,180,181,182,183,184,185,186,187,188,189,190,191,192,193,194]. These include sudies of gray matter volume (GMV), cortical thickness, diffusion tensor imaging, dorsal nigral hyperintensity (DNH), neuromelanin-sensitive structural and diffusion MRI, and functional MRI.

### 5.1. Studies on Gray Matter Volume (GMV)

Most studies on GMV have used voxel-based morphometry. Although several of these studies have not shown significant clusters of reduced GMV or similar total brain volumes in between iRBD patients compared with controls [164,178,189], others have shown non-homogeneous results in iRBD patients, including smaller bilateral putamen volumes [164], decreased GMV in the anterior lobes of the right and left cerebellum, tegmental portion of the pons, and left parahippocampal gyrus [166], decreased GMV in the frontal lobes, anterior cingulate gyri, and caudate nucleus [172], decreased GMV in the frontal, temporal, parietal, occipital cortices as well as increased GMV in cerebellum posterior lobe, putamen, and thalamus [182], decreased GMV in the brainstem, anterior cingulate and insula [191], decreased GMV of the right caudate nucleus [194], and right hippocampal atrophy [177]. Bourgouin et al., 2019 [176] described reduced GMV in the caudate nucleus of iRBD patients with depressive symptoms and in the left amygdala-hippocampus in iRBD patients with anxiety symptoms when compared to iRBD patients without these symptoms and with controls. In contrast, other studies have shown increases of GMV in both hippocampi [165], or the frontal cortex, thalamus, and caudate nucleus of iRBD patients [175].

Compared with PD patients without RBD and controls, patients with concomitant PD and RBD or probable RBD have shown decreased volumes in the pontomesencephalic tegmentum (location of cholinergic, GABAergic, and glutamatergic neurons), medullary reticular formation, hypothalamus, thalamus, putamen, amygdala, and anterior cingulate cortex [171], volume decrease over time in the left caudate nucleus, pallidum and amygdale [187], and a relatively high GMV in the cerebellar vermis IV/V and low GMV in the right superior occipital gyrus [188].

### 5.2. Studies on Cortical Thickness

Compared with controls, iRBD patients showed, according to Rahayel et al. [172] thinning in bilateral anterior cingulate, orbitofrontal, and medial superior frontal cortices, and in the right dorsolateral primary motor cortex. Patients without MCI showed cortical thinning in the frontal cortex only, while those with cognitive impairment showed cortical thinning in the cingulate, temporal, frontal, and occipital cortices, and abnormal surface contraction in the thalamus and in the lenticular nucleus [174].

Campabadal et al. [177] described cortical thinning in the left superior parietal, post-central, and fusiform regions, and right superior frontal and lateral occipital regions, and significant differences between iRBD patients and controls in the progression of cortical thinning in the left occipital pole and lateral orbitofrontal gyri the right cuneus, and bilateral superior parietal lobe and precuneus [184]. Pereira et al. [178] reported parietal and occipital cortical thinning in iRBD patients compared to controls, and cortical thinning in frontal, occipital, and parietal areas in iRBD patients who developed further neurodegenerative disease compared to iRBD non-converters.

Compared with PD patients without RBD, patients with PD and RBD or probable RBD showed bilateral inferior temporal cortex thinning, and an increase in the rate of thinning in the left insula during a follow-up period [187].

### 5.3. Studies Using Diffusion Tensor Imaging (DTI)

While one study showed significant microstructural changes in the white matter at several areas including the brainstem, the right substantia nigra, the left temporal lobe, the olfactory region the fornix, the corona radiata, the internal capsule and the right visual stream of iRBD patients compared with controls [163], other failed to find any microstructural difference between iRBD patients, prodromal PD patients, and controls, although described a significant increase in mean diffusivity (MD) in prodromal PD relative to iRBD in widespread, but mostly right-lateralized regions [179]. A study described decreased fractional anisotropy (FA) in the rostral pons and in the tegmentum of the midbrain and increased MD within the pontine reticular formation overlapping with a cluster of decreased FA in the midbrain in iRBD patients [90], and other decreased in FA, and no differences in axial diffusivity (AD), radial diffusivity (RD), or MD in the SN in iRBD patients compared with controls [173].

Finally, a study of free-water maps with a bi-tensor model showed higher free-water values in the posterior SN in patients with iRBD (which increased over time) than in controls, but significantly lower than in patients with PD [193].

### 5.4. Studies Addressing Dorsolateral Nigral Hyperintensity (DNH)

Two studies addressed the measurement of DNH by using high-field susceptibility-weighted imaging (SWI). One of them described that at least two-thirds of iRBD individuals showed loss of DNH, a frequency which was similar to that observed in PD and higher than that found in controls [170], while other showed loss of DNH in only 27.5% of RBD patients (and 7.7% of control subjects and 96% of PD patients) [35]. It is possible that loss of DNH could be useful in identify prodromal degenerative parkinsonism in iRBD.

### 5.5. Studies with Neuromelanin-Sensitive Structural and Diffusion MRI

Studies using this method showed reduced signal intensity in the locus *coeruleus*/*subcoeruleus* complex [169] and in the SN [173], in iRBD patients compared with controls. PD patients also have shown reduced signal intensity in the locus *coeruleus/subcoeruleus* area compared with controls, which was more marked in patients with coexistent RBD [167].

### 5.6. Studies with Functional MRI (fMRI)

The first study with resting-state fMRI (rsfMRI) showed decreased nigrostriatal and nigrocortical connectivity (between SN and right cuneus/precuneus and superior occipital gyrus) in iRBD patients compared with controls but increased connectivity compared with PD patients [168]. Further studies have shown the following findings:

1. Decreased functional connectivity in the basal ganglia network both in iRBD and in PD patients which differentiated iRBD and PD from controls with high sensitivity and specificity, but did not differentiate RBD from controls [28].
2. Reduced cortico-cortical functional connectivity strength in edges located in posterior regions in iRBD patients compared with controls [180].
3. Increased functional connectivity between the left thalamus and occipital regions including the right cuneal cortex, left fusiform gyrus, and lingual gyrus in iRBD patients compared with controls [181].
4. Decreased functional connectivity between the limbic striatum and temporo-occipital regions in iRBD patients compared with controls, which was associated with the presence of impulse control disorder in iRBD patients [189].
5. Decreased functional connectivity between the brainstem and the anterior cingulated, temporal lobe, and the cerebellum posterior lobe in iRBD patients compared with controls [191].
6. Decreased striatal-prefrontal (this in executive control) and midbrain-pallidum functional connectivity in the basal ganglia network, decreased motor and somatosensory cortex functional connectivity in the sensorimotor network, and lack of abnormalities in the default mode network compared with controls [190].
7. Decreased functional connectivity with two clusters located in the precuneus in iRBD patients compared to the controls using a multivariate pattern analysis [186].

A study on structural connectivity showed decreased measures of the global network, average degree, global efficiency, and local efficiency, and increased measures of characteristic path length in iRBD patients compared to controls [175]. Two studies measuring amplitude of low-frequency fluctuations (ALFF), showed, respectively, increased ALFF values in the right parahippocampal gyrus [182], and normal values [36]. A significant reduction in regional homogeneity in the bilateral putamen of iRBD patients compared with controls has also been described [36].

Finally, two studies addressed the possible differences in functional connectivity (one of them in dynamic functional connectivity) between PD patients with and without concomitant RBD. One of them showed decreased functional connectivity between the right superior occipital gyrus and the posterior regions (left fusiform gyrus, left calcarine sulcus, and left superior parietal gyrus [188], and the other a brain pattern mainly marked by weaker positive couplings between visual network and default mode network, default mode network and basal ganglia network, and within default mode network [192], in PD patients with RBD compared with those without RBD.

### 5.7. Measurement of Visible Enlarged Perivascular Space (EPVS)

A recent study described higher burdens of EPVS in the centrum semiovale, basal ganglia, substantia nigra, and brainstem of iRBD patients in comparison with PD patients and controls, being higher EPVS in the centrum semiovale and the brainstem as independent risk factors for iRBD [185].

## 6. Conclusions

There are enough data suggesting the role of dopaminergic dysfunction in iRBD. Data from functional neuroimaging studies with PET/SPECT have shown that many patients diagnosed, at least initially, with iRBD, had decreased uptake of tracers for the presynaptic dopaminergic terminal, suggesting the presence of preclinical parkinsonism, and, moreover, many of the patients develop PD or other synucleopathies later. The high frequency of SNH in patients with iRBD is consistent with these data, and the combination of functional neuroimaging studies with TCS has shown to be useful to predict the phenoconversion of iRBD to a synucleopathy [21].

The possible role of noradrenaline is supported by functional neuroimaging studies with noradrenaline ligands showing decreased uptake in the primary sensorimotor cortex [73] and in the thalamus [69], and the finding of ^123^I-MIBG in iRBD patients [69,83,84,85,86,87,88,89,90,91,92,93,94,95], a finding that also appears consistently in PD and DLB patients. The finding of cell loss in the *locus ceruleus* in a post-mortem study [79] is consistent with noradrenergic deficiency, but it is limited to a single patient.

Despite induction of RBD by antidepressants suggest a role of serotonin in this condition [77,78,97,98,99,100,101,102,103,104], data from functional neuroimaging studies do not support a role of this neurotransmitter in RBD [25,46], although serotonine could be related to the presence of apathy in some patients [32].

Data from functional neuroimaging studies showing a decreased uptake of the acetylcholine transporter in the brainstem [74] and decreased uptake of tracers of the postsynaptic cholinergic terminals in several cortical areas [68,75], and in the colon [69], suggest a possible role of acetylcholine in the pathophysiology of iRBD. In addition, the results of studies on SAI of the motor cortex are consistent with the presence of a cholinergic deficit in patients with iRBD and with RBD associated with PD [112,113], giving support to this hypothesis. In contrast, the results of measurements of choline/creatinine ratio in ^1^H-MRS studies have not shown conclusive results.

The peptide ghreline, the hormone melatonin, proinflammatory markers such as IL-10 and TNF-α, several lipoproteins and glycosylated proteins, and specially alpha-synuclein, could be considered as potentially interesting markers for RBD and to predict its possible phenoconversion to synucleopathies.

In general, brain perfusion studies and brain glucose metabolism studies have shown increased cerebral blood flow and glucose metabolism in the pons, thalamus, hippocampus, and cerebellum, decreased cerebral blood flow in the temporoparietal cortex and decreased cortical metabolic activity with predominance in temporoparietal, prefrontal, occipital, and supplementary motor area, a pattern resembling that of PD and LBD.

The results of structural and fMRI are still controversial (Table 4). Most of them have shown the presence of structural changes in deep gray matter nuclei, cortical gray matter atrophy, and alterations in the functional connectivity within the basal ganglia, the cortico-striatal, and the cortico-cortical networks [195]. However, there is a lack of homogeneity of both the methods used and the results obtained, and many of these studies were based on low sample sizes.

The design of prospective multicenter studies involving a large series of iRBD patients and controls with a long-term follow-up period, using multiple biochemical and multimodal neuroimaging parameters should be appropriate in an attempt to establish the neurochemical features of iRBD and the risk factors for its phenoconversion to synucleopathies.

## Figures and Tables

**Table 2:** Studies assessing cerebral blood flow/brain perfusion in patients with RBD. BA Brodmann areas; iRBD idiopathic RBD; LBD Lewy body dementia; MCI mild cognitive impairment; MRI magnetic resonance imaging; pASL Pseudocontinuous arterial spin-labeled; PD Parkinson’s disease; RBD REM sleep behavior disorder; ^99m^Tc-ECD SPECT: ^99m^Tc-Ethylene Cysteinate Dimer single-photon emission computerized tomography; ^99m^Tc-HMPAO SPECT: ^99m^Tc-hexamethylpropyleneamine oxime single-photon emission computerized tomography.

Authors, Year [Ref]	Patients/Controls	Method	Main Findings
Mazza et al., 2006 [146]	9/8	^99m^Tc-ECD SPECT	Increased perfusion in the pons and putamen bilaterally and in the right hippocampus Decreased perfusion in the temporo-parietal (left BA 7, 19, 20, 21, 39, 40, 41, and 42, and bilateral BA 12, 22, and 44) and in frontal cortices (left 9 and 46 BA and bilateral BA 4, 6, 10, 43, 44, and 47).
Hanyu et al., 2011 [147]	24/18	N-isopropyl-p-^123^I-iodoamphetamine SPECT	Decreased regional cerebral blood flow in the parietooccipital lobe (precuneus) and cerebellar hemispheres
Vendette et al., 2011 [148]	20/20	^99m^Tc-ECD SPECT	Increased regional cerebral blood flow in bilateral pons, putamen, and hippocampus. Decreased cerebral blood flow in subcortical regions in medial parietal areas. Association between brain perfusion in the frontal cortex and occipital areas with poorer performance in color discrimination test. Relationship between cerebral blood flow decrease in the bilateral anterior parahippocampal gyrus and loss of olfactory discrimination These patterns are similar to those seen in PD and LBD.
Vendette et al., 2011 [149]	20/20 (10 RBD patients had mild cognitive impairment- MCI)	^99m^Tc-ECD SPECT	Both subgroups of RBD patients showed hypoperfusion in the frontal regions, right hippocampus, and parahippocampal gyri. RBD patients with MCI showed cortical hypoperfusion in the occipital, temporal, and parietal regions compared with RBD patients without MCI and with controls. RBD patients with MCI showed more pronounced alterations in the right hippocampus and had increased perfusion in the putamen and on the left paracentral gyrus. These patterns are similar to those seen in PD and LBD.
Dang-Vu et al., 2012 [150]	20/0 (10 RBD patients developed PD or LBD after 3 years of follow-up)	^99m^Tc-ECD SPECT	Increased regional cerebral blood flow in the hippocampus of patients who converted to PD or LBD compared with those who did not. Correlation between hippocampal perfusion with motor and color vision scores in RBD patients.
Sakurai et al., 2014 [151]	9/0	N-isopropyl-p-^123^I-iodoamphetamine SPECT	Follow-up study of 9 patients of cohort described in reference [22] 8 months later [149]. Decrease in regional cerebral blood flow in bilateral parietotemporal and occipital areas in the two studies. Decrease in regional cerebral blood flow in the medial portions of the parietooccipital lobe with a significant decrease in the right posterior cingulated (compared with the 1st SPECT)
Mayer et al., 2015 [152]	1/0 (+1 PD with RBD and 2 narcolepsy with RBD)	^99m^Tc-ECD SPECT	Ictal single-photon emission tomography displayed the same activation in the bilateral premotor areas, the interhemispheric cleft, the periaqueductal area, the dorsal and ventral pons, and the anterior lobe of the cerebellum in all patients
Chen et al., 2020 [153]	15/20	Pseudocontinuous arterial spin-labeled (pASL) perfusion with high resolution T1-weighted images using a 3.0 Tesla MRI unit	Patients with iRBD showed decreased regional cerebral blood flow values in the right inferior frontal gyrus, right middle frontal gyrus, and right insula This pattern is similar to that seen in PD and LBD.
Barill et al., 2020 [154]	37/23	^99m^Tc-HMPAO SPECT	Patients with iRBD showed decreased regional perfusion in the anterior frontal and lateral parietotemporal cortex compared with controls. A second SPECT performed 17 months later in iRBD patients showed a relative increase (with reversion to normal levels in controls) in regional perfusion in the anterior frontal, lateral parietal, and occipitotemporal cortices
Eskildsen et al., 2021 [155]	20/25	Dynamic susceptibility contrast MRI using spin echo echo-planar imaging with high resolution T1-weighted images using a 3.0 Tesla MRI unit	Patients with iRBD showed profound hypoperfusion and microvascular flow disturbances throughout the cortex in patients compared to controls, especially in cortical areas related to language comprehension, visual processing, and recognition. Cortical hypoperfusion was associated with impaired cognitive performance.

**Table 3:** Studies assessing cerebral glucose metabolism in patients with RBD. AD Alzheimer’s disease; BA Broddmann areas; ^11^C-CFT: 2b-carbomethoxy-3b- (4-trimethylstannylphenyl) tropane; DAT dopamine transporter; dnPDRBD-RP de novo PD RBE related pattern; EMG electromyography; ^18^F-FDG: ^18^F-fluoro-d-glucose; ^18^F-FP-CIT: ^18^F-N-(3-fluoropropyl)-2beta-carbon ethoxy-3beta-(4-iodophenyl) nortropane;iRBD-AB idiopathic RBD with abnormal DAT scan; iRBD-RN idiopathic RBD with relatively normal DAT scabn; iRBD-RP idiopathic RBD related pattern; LBD Lewy body dementia; MCI: mild cognitive impairment; PD Parkinson’s disease; PDRP PD related pattern; PPDRBDRP PD-RBD related pattern; PET: Positron Emission Tomography; RBD REM sleep behavior disorder; SPECT: Single Photon Emission Computerized Tomography; VOI volumes of interest.

Authors, Year [Ref]	Patients/Controls	Method	Main Findings
Fujishiro et al., 2010 [92]	9/0	^18^F-FDG PET	Compared to the normative database, 4 of 9 patients showed diffuse areas of glucose hypometabolism, with a predominance in the occipital lobe (similarly to patients with LBD), which was related with scores in task of visuospatial ability. The 5 patients without occipital hypometabolism showed (similarly to PD patients) hypometabolism in the right anterior temporal lobe (BA 38), right frontal lobe (BA 32), and left anterior cingulate gyrus (BA 24)
Wu et al., 2014 [156]	21/21 (and 16 moderate PD patients)	^18^F-FDG PET	iRBD and PD patients showed, in comparison to controls, increased glucose metabolism in the pons, thalamus, posterior cerebellum, supramarginal, and inferior temporal gyri, hippocampus, medial frontal and sensorimotor areas, and, decreased metabolism in superior temporal and occipital regions. The previously described alterations decreased with the progression of the disease.
Wing et al., 2015 [41]	11 (with comorbid major depressive disorder)/10 (+8 with major depressive disorder without RBD)	^18^F-FDG PET	No significant differences in the striatal tracer uptake among the 3 study groups.
Ge et al., 2015 [157]	20/21	^18^F-FDG PET	Compared with controls, patients with RBD showed decreased metabolism in the occipital cortex/lingual gyrus, and increased metabolism in the pons, cingulate, supplementary motor area, and hippocampus/parahippocampus RBD duration correlated with metabolism positively in the anterior vermis, and negatively in the medial frontal gyrus. Chin EMG activity presented a positive metabolic correlation in the hippocampus/parahippocampus, and a negative metabolic correlation in the posterior cingulated.
Ota et al., 2016 [158]	11 iRBD non-demented with cognitive decline 4 years later	^18^F-FDG PET	Four patients developed LBD, 3 developed PDD, and 4 developed cognitive decline without clinical features of LBD. Patients who developed LBD and cognitive decline showed decreased glucose metabolism in the occipital lobe (more marked in the primary visual cortex). Patients who developed LBD A showed a trend to hypometabolism in the parieto-temporal cortex. Patients of group who developed cognitive decline showed a trend toward hypometabolism in the anterior cingulated gyrus and in the medial prefrontal area
Meles et al., 2017 [29]	21/19 (+20 PD and 22 DLB patients)	^18^F-FDG PET and DAT. Analysis of PD-related pattern (PDRP) expression (characterized by relatively decreased cortical glucose metabolism in the supplementary motor area, and in the prefrontal association, posterior and inferior parietal, lateral occipital, and temporal cortices, and a relative increased metabolism in the pons, cerebellum, pallidum, thalamus, limbic association and sensorimotor cortices, left supplementary motor area, and paracentral lobule)	iRBD subjects showed higher PDRP expression than controls, but lower than that of PD and LBD. iRBD subjects with hyposmia and/or abnormal DAT scan showed higher PDRP expression
Meles et al., 2017 [159]	21/44 (+38 de novo PD patients; 24 with probable RBD)	^18^F-FDG PET and DAT. Analysis of iRBD related pattern (iRBDRP; relative increased glucose metabolism in the brain stem cerebellum, thalamus, hippocampus, and sensorimotor cortex, and decreased metabolism in the middle cingulated, parietal, temporal, and occipital cortices) which showed a partial overlapping with the PDRP.	iRBDRP was significantly expressed in PD patients compared with controls iRBDRP expression was not significantly different between PD patients with and without probable RBD, or between PD patients with unilateral or bilateral parkinsonism. iRBDRP) expression was higher in PD with MCI patients than in PD patients with preserved cognition. Subject scores on the iRBDRP were highly correlated to subject scores on the PDRP. Expression of both PDRP and iRBDRP was higher in patients with a more severe form of PD, which indicates that expression of the 2 patterns increases with disease severity.
Huang et al., 2020 [72]	37/15 (+86 PD patients)	^18^F-FDG PET and ^11^C-CFT PET. Patients were divided into those with relatively normal (iRBD-RN) or abnormal (iRBD-AB) striatal DAT binding	Significant but modest correlations between DAT binding and PDRP expression in the iRBD-AB and PD groups but not in the iRBD-RN group. iRBD patients presented with an intermediate state in PDRP activity between PD and normal controls. Correlations between these two measures, both in iRBD and in PD patients, sugges that nigrostriatal dopaminergic denervation alone does not explain completely the differences in network activity
Arnaldi et al., 2019 [160]	36/79 (+72 PD patients; 40 with probable RBD)	^18^F-FDG PET. Discriminant analysis according to the metabolic pattern	The model confounded partially iRBD and PD without RBD, although was moderately accurate in discriminating the correct category between iRBD, PDRBD, PD without RBD, and controls
Yoon et al. [45]	28/24 (+21 patients with PDRBD)	^18^F-FDG PET and ^18^F-FP-CIT PET. Analysis of PDRBDRP	PDRBDRP showed a relative increased glucose metabolism in the premotor cortex and hippocampus, and also reflected the PD-related covariance pattern. reported previously PDRBDRP was significantly higher in PDRBD patients than in iRBD patients, and in iRBD patients than in controls. Negative correlation of PDRBDRP with olfactory and frontal executive functions and with the striatal DAT density. 5 of 11 iRBD patients with PDRBDRP elevation (and none patient without PDRBDRP) developed Lewy body diseases after 3.5 years of follow-up.
Liguori et al. [161]	54/35 (+28 patients with PD, 10 with LBD and 55 with Alzheimer disease -AD)	^18^F-FDG PET	iRBD patients showed increased 18F-FDG uptake in the brainstem and in several areas of the temporal lobe, limbic lobe and frontal lobe, and reduced 18F-FDG uptake in temporal and parietal regions compared to controls. iRBD patients showed several differences in 18F-FDG uptake when compared with PD (increased uptake in the midbrain and pons, cerebellum, lentiform nucleus, claustrum, and frontal and temporal lobes), LBD (relative hypometabolism in frontal and limbic lobes), or AD groups, being the comparison with AD patients that which showed the main differences (reduced uptake in the frontal lobe and increased uptake in limbic, temporal and parietal lobes).
Mattioli et al., 2021 [37]	39 (17 with MCI)/42	^18^F-FDG-PET and ^123^I-FP-CIT-SPECT	iRBD patients with MCI showed a relative hypometabolism involving precuneus and cuneus which was correlated with verbal memory, executive functions, and nigro-putaminal DAT binding. Interregional correlation analysis (used to explore brain functional connectivity), showed a brain functional network in iRBD patients involving regions in occipital, temporal, parietal, and frontal lobes, caudate nuclei, thalamus, and red nuclei (this network was smaller in controls and involved cingulate, occipital, and temporal cortices, and cerebellum). MCI-VOI was involved in a white matter network including corpus callosum and cingulate fasciculus.
Shin et al., 2021 [162]	30/24 (+28 patients with de novo PD and RBD –dnPDRBD-, and 21 with PD without RBD)	^18^F-FDG-PET. Calculation of derived metabolic patterns from the PDRP and dnPDRBDRP scores in iRBD patients.	Future phenoconversion of iRBD patients was predicted, with all cut-off ranges, by high dnPDRBDRP. The predictability of PDRP future phenoconversion only was significant in a partial range of cut-off.

**Table 4:** Results of structural and functional neuroimaging in patients with REM sleep behavior disorder (RBD). AD axial diffusivity; ALFF amplitude of low-frequency fluctuations; BG basal ganglia; BOLD blood oxygen level-dependent; BS brainstem; CSO centrum semiovale; DAT dopamine transporter; DBM deformation-based morphometry; DFC dynamic functional connectivity; DNH dorsal nigral hyperintensity; DTI diffusion tensor imaging; EPVS enlarged perivascular space; FA fractional anisotropy; fMRI functional MRI; GE-EPI gradient echo planar imaging; GM gray matter; GMD gray matter density; GMV gray matter volume; iRBD idiopathic RBD; MCI mild cognitive impairment; MD mean diffusivity; MRI magnetic resonance imaging; MVPA multivariate pattern analysis; NM neuromelanin; PD Parkinson’s disease; RBD REM sleep behavior disorder: RD radial diffusivity; ReHo regional homogeneity; SN substantia nigra; SWI susceptibility-weighted imaging; T Tesla; TIV total intracranial volume; VBM voxel-based morphometry.

Authors, Year [Ref]	RBD/ Controls	Method	Main Findings
Unger et al., 2010 [163]	12/10	1.5 T MRI single-shot echo planar sequence with a twice-refocused spin echo pulse, sequence acquisition, processed with voxel-wise analysis of DTI. Measurement of AD (a potential marker of neuronal loss), RD (a potential marker of glial pathology), and FA (measure of brain-tissue integrity)	The white matter of the brainstem, the right substantia nigra, the left temporal lobe, the olfactory region, the fornix, the corona radiata, the internal capsule, and the right visual stream, showed significant microstructural changes in patients with iRBD. These regions are involved in REM-sleep regulation and neurodegenerative pathology in iRBD and/or early PD
Ellmore et al., 2010 [164]	5/17 (+5 early PD patients)	3.0 T MRI T1-weighted acquisition and processed with VBM. Quantification of volumes of specific subcortical gray matter nuclei implicated in PD	Total brain volumes were similar in RBD, PD, and controls RBD group showed smaller bilateral putamen volumes
Scherfler et al., 2011 [165]	26/14	1.5 T MRI T1-weighted acquisition and processed with VBM and DTI. Measurement of MD and FA	The tegmentum of the midbrain and rostral pons showed a significant decrease of FA, and the pontine reticular formation showed increase of MD in iRBD patients (these overlapped with a cluster of decreased FA in the midbrain). Increases of GMD in both hippocampi of iRBD patients.
Hanyu et al., 2012 [166]	20/18	1.5 T MRI T1-weighted acquisition and processed with VBM.	GMV in the tegmental portion of the pons, anterior lobes of the both cerebellar hemispheres, and left parahippocampal gyrus was significantly reduced in patients with iRBD
García-Lorenzo et al., 2013 [167]	36 PD patients (24 of them with RBD)/19	3.0 T MRI T1-weighted acquisition, and processing by using combined NM-sensitive, structural and diffusion MRI approaches	When compared with controls, PD patients (specially those with RBD) showed decreased signal intensity in the locus coeruleus/subcoeruleus area. The percentage of increase in abnormal muscle tone during REM sleep was correlated with reduced signal intensity
Ellmore et al., 2013 [168]	10/10 (+11 PD patients)	3.0 T MRI T1-weighted acquisition. Measurement of correlations of SN time series using resting-state BOLD-fMRI and voxelwise analysis of variance	Patients with RBD showed different correlations between left SN and left putamen in comparison with controls and patients with PD. SN correlations with superior occipital gyrus and right cuneus/precuneus were significantly different for RBD patients with RBD compared with PD patients and with controls. These results suggest the presence of altered nigrostriatal and nigrocortical connectivity in RBD patients before the onset of obvious motor impairment
Ehrminger et al., 2016 [169]	21/21	3.0 T MRI T1-weighted acquisition, and processing by using NM-sensitive imaging	iRBD patients showed reduced signal intensity in the locus coeruleus/subcoeruleus complex The mean sensitivity and specificity of the visual analyses of the signal for the identification of iRBD were 82.5% and 81%
De Marzi et al., 2016 [170]	15/42 (+104 PD patients)	3.0 T MRI T1-weighted acquisition. Measurement of DNH with high-field SWI	At least two-thirds of iRBD patients showed (this frequency approaches to the rate observed in PD and higher than that found in controls). According to the authors the absence of DNH could be a useful tool to identify prodromal degenerative parkinsonism in patients with iRBD.
Boucetta et al., 2016 [171]	309 PD patients (69 of them with probable RBD)/19	3.0 or 1.5 T MRI T1-weighted acquisition and processed with DBM	PD patients with probable RBD showed smaller volumes than patients without RBD and controls in the pontomesencephalic tegmentum, medullary reticular formation, hypothalamus, thalamus, putamen, amygdala, and anterior cingulate cortex. These results suggest an association of RBD with loss of volume in the pontomesencephalic tegmentum (where cholinergic, GABAergic, and glutamatergic neurons coexist).
Rolinski et al., 2016 [28]	26/23 (+48 early PD patients)	3.0 T MRI. T1-weighted acquisition, and processing with VBM. Measurement of basal ganglia network dysfunction by using resting-state fMRI	Connectivity measures of basal ganglia network dysfunction differentiated both RBD and PD from controls with high sensitivity (96%) and specificity (74% for RBD and 78% for PD). RBD was indistinguishable from Parkinson’s disease on resting-state fMRI.
Rahayel et al., 2018 [172]	41/41	3.0 T MRI T1-weighted acquisition and processed with VBM.	Cortical thickness analysis showed thinning in iRBD patients in the anterior cingulate orbitofrontal bilateral medial superior and frontal cortices of both hemispheres, and in the right dorsolateral primary motor cortex. iRBD patients showed decreased GMV in the anterior cingulate gyri, frontal lobes, and caudate nucleus. Extensive surface contraction in the internal and external segments of the left pallidum was shown with shape analysis Clinical and motor-impaired features in iRBD were associated with all these anomalies of the motor cortico-subcortical loop.
Frosini et al., 2015 [65]	15/14 (+28 PD patients)	7.0 T MRI T1-weighted acquisition. Processing by using three-dimensional gradient-recalled-echo multi-echo SWI of the SN	Nine subjects with RBD had abnormal SPECT; among them, the findings of 7T-MRI were rated abnormally in eight. Normal 7T-MRI findings of five out of six subjects with RBD with normal SPECT.
Pyatigorskaya et al., 2017 [173]	19/18	3-T MRI, with analysis of DTI, NM-sensitive imaging, and T2* mapping. Regions of interest in the SN area were drawn in NM-sensitive and T2-weighted images	Compared with controls, iRBD patients showed a reduction in the NM-sensitive volume and signal intensity and a decrease in FA, and no differences in AD, RD, or MD or in R2*. The receiver operating characteristic analysis of NM-sensitive volume and signal intensity was able to discriminate between iRBD patients and controls with a high diagnostic accuracy. These three biomarkers had a combined accuracy of 0.92.
Rahayel et al., 2018 [174]	52 (17 with MCI)/41	3.0 T MRI T1-weighted acquisition and processed with VBM.	Patients with MCI showed cortical thinning in the occipital, temporal, frontal, and cingulate cortices, and abnormal surface contraction in the thalamus and in the lenticular nucleus. Cortical thinning in patients without MCI is restricted to the frontal cortex. Association between lower performance in cognitive domains and cortical and subcortical abnormalities. Association of impaired performance on olfaction, color discrimination, and autonomic measures with occipital lobe thinning.
Park et al., 2019 [175]	10/14	3.0 T MRI T1-weighted acquisition, calculation of absolute structural volumes using FreeSurfer image analysis software, and structural volume and connectivity analyses performed with Brain Analysis using Graph Theory.	Compared to healthy controls, patients with iRBD showed significantly increased volumes of the frontal cortex, thalamus, and caudate nucleus. When compared to controls, iRBD patients showed significantly different structural connectivity (decreases in measures of the global network, global efficiency, local efficiency and average degree; and increase in characteristic path length; and significant hub reorganization in measures of the local network). Increased betweenness centrality of the caudate nucleus and frontal cortex in iRBD patients.
Bourgouin et al., 2019 [176]	46/31	3.0 T MRI T1-weighted acquisition and processed with VBM.	iRBD patients with depressive symptoms showed reduced GMV in the caudate nucleus compared to controls and iRBD patients without depressive symptoms. iRBD patients with anxiety symptoms showed reduced GMV in the left amygdala extending to the hippocampus compared to controls and iRBD patients without anxiety symptoms. Higher scores for depression and anxiety in iRBD patients were associated with lower GMV in these regions respectively.
Campabadal et al., 2019 [177]	20/27	3.0 T MRI T1-weighted acquisition; FreeSurfer was used to estimate cortical thickness, subcortical volumetry, and hippocampal subfields segmentation; and FIRST, FSL’s model-based segmentation/registra-tion tool to perform hippocampal shape analysis	iRBD patients showed impairment in attention, verbal naming, verbal memory, facial recognition, and processing speed, in comparison with controls. iRBD patients had cortical thinning in right superior frontal and lateral occipital regions, and in left post-central, superior parietal, and fusiform regions. iRBD patients showed right hippocampal atrophy (specifically in posterior regions) in volumetric and shape analyses. iRBD patients showed significant differences in the right CA1, molecular layer, granule cell layer of the dentate gyrus, and CA4, in the hippocampal subfields exploratory analysis. Lack of correlation between cognitive performance and brain atrophy.
Pereira et al., 2019 [178]	27/31 (+ 151 de novo PD patients)	3.0 T MRI T1-weighted acquisition. Cortical thickness and volumes of subcortical gray matter structures (hippocampus, amygdala, thalamus, caudate, putamen, pallidum, accumbens) processed with Freesurfer39 in addition to the estimated total intracranial volume (TIV)	At baseline, iRBD patients showed parietal and occipital cortical thinning, compared to controls. They also showed worse motor and non-motor abilities, some of which correlated with motor, frontal or temporal cortical thinning. At 3-years of follow-up, 22% iRBD patients were diagnosed with a Lewy body disorder. These patients showed cortical thinning in frontal, occipital, and parietal areas compared to iRBD non-converters. The future development of a Lewy body disorder was predicted by the presence of cortical thinning. Subcortical gray matter was similar between groups
Yamada et al., 2019 [33]	23 (8 with mild motor impairment)/20	3.0 T MRI T1-weighted acquisition resting state whole-brain fMRI acquired with single-shot gradient echo planar imaging (GE-EPI).	When compared to iRBD patients with normal motor function and with controls, iRBD patients with mild motor impairment showed increased cortico-cerebellar functional connectivity and decreased cortico-striatal functional connectivity
Ohlhauser et al., 2019 [179]	17 (all converted further to PD)/21 (+20 prodromal PD patients, 14 with RBD and 6 with hyposmia)	3.0 T MRI T1-weighted acquisition, and processed with DTI. Measurement of MD and FA by using tract-based spatial statistics	Significant increased in MD in prodromal PD relative to iRBD in widespread, but mostly right-lateralized regions (this pattern was particular to individuals with RBD). Lack of microstructural differences between controls, prodromal PD, and iRBD patients. The prodromal PD group had significantly higher RBD symptoms and the iRBD group had significantly higher motor symptoms.
Barber et al., 2020 [35]	46/32 (+28 PD patients)	3.0 T MRI T2*-weighted images T1-weighted acquisition and T1-weighted structural MRI acquisition; assessment of DNH using SWI MRI.	The frequencies of DNH in RBD patients, controls, and PD patients were, respectively, 27.5%, 7.7%, and 96%. Mean quantified DNH signal intensity in RBD patients was lower than that of controls, and higher than that of PD patients
Campabadal et al., 2020 [180]	20/27	3.0 T MRI T1-weighted acquisition; study of brain functional connectivity using resting-state fMRI	Compared with controls, iRBD patients showed reduced cortico-cortical functional connectivity strength in edges located in posterior regions (this regional pattern was also shown in an independent analysis comprising posterior areas where a decreased connectivity in 51 edges was found, whereas no significant results were detected in the anterior network). iRBD patients showed reduced centrality of the left superior parietal lobule in the posterior network. iRBD patients, but not controls, showed a positive correlation between functional connectivity strength in the right superior parietal and left inferior temporal lobes with mental processing speed
Byun et al., 2020 [181]	37/15	3.0 T MRI T1-weighted acquisition; resting-state fMRI and seed-to voxel analysis were used to study thalamo-cortical functional connectivity	iRBD patients showed higher functional connectivity than controls between the left thalamus and occipital regions (these included the left fusiform gyrus, and lingual gyrus, and the right cuneal cortex). Thalamo-fusiform functional connectivity was positively correlated with word list recognition
Chen et al., 2020 [182]	27/33	3.0 T MRI T1-weighted acquisition; VBM was used to assess gray matter alterations, with VBM; resting-state fMRI to study thalamo-cortical functional connectivity, and calculation ALFF to measure differences in spontaneous brain activity	Compared with healthy controls, patients with iRBD showed decreased GMV in the frontal, temporal, parietal, occipital cortices as well as increased grey matter volume in cerebellum posterior lobe, putamen, and thalamus. Patients with iRBD showed increased ALFF values in the right parahippocampal gyrus. In the occipital cortices ALFF value changes were correlated with olfaction.
Ellmore et al., 2020 [183]	32/11 (+23 PD patients, 16 with RBD)	3.0 T MRI T1-weighted acquisition; study of the whole brain SN functional connectivity using resting-state fMRI and voxelwise analysis; correlation with serum uric acid levels	Controls showed a positive relationship between uric acid and SN functional connectivity with the left lingual gyrus. This was negative in PD patients and was reduced in patients with RBD and with PD and RBD
Li et al., 2020 [36]	15/20	3.0 T MRI T1-weighted acquisition; measurement of spontaneous neuronal activity of the striatum ReHo and ALFF analysis	Significant reduction in ReHo in the bilateral putamen of iRBD patients, compared with controls iRBD patients showed a positive correlation between the mean ReHo value and the DAT tracer uptake ratio in the left putamen.
Campabadal et al., 2020 [184]	14/18	3.0 T MRI T1-weighted acquisition; estimation of cortical thickness and subcortical volumetry with FreeSurfer	iRBD patients and controls showed significant differences in cortical thinning progression in bilateral precuneus and superior parietal areas, in the left occipital pole and lateral orbitofrontal gyri, and in the right cuneus Worse progression in the visual form discrimination test in iRBD patients was associated with loss of gray matter in the left precuneus and in the right superior parietal area. Cortical thinning (specially in frontal regions) was associated with increased motor signs in iRBD patients. Cortical thinning involving posterior areas was associated with late-onset iRBD.
Si et al., 2020 [185]	33/35 (+82 PD patients, 39 with RBD)	3.0 T MRI T1-weighted acquisition; assessment of visible EPVS in CSO, BG, SN, and BS	iRBD patients had significantly higher EPVS burdens (CSO, BG, SN, and BS) than PD patients and controls. Higher BS-EPVS and CSO-EPVS burdens were strongly associated with the risk for iRBD. Higher SN-EPVS and CSO-EPVS were correlated with the severity iRBD symptoms. Higher BG-EPVS burden was correlated with cognitive impairment scores in PD patients.
Byun et al., 2020 [186]	50/20	3.0 T MRI T1-weighted acquisition; use of resting-state fMRI and MVPA to study whole-brain functional connectivity	MVPA showed a significant cluster in the left posterior insular cortex. iRBD patients showed, compared to the controls, significantly reduced functional connectivity with two clusters located in the precuneus. using the cluster as a seed in a seed-to-voxel functional connectivity analysis Association between the degree of functional connectivity and cognitive scores
Yoon et al., 2021 [187]	78 PD patients (18 with probable RBD)	3.0 T MRI T1-weighted acquisition; comparison between PD patients with RBD and without RBD of both cross-sectional and longitudinal cortical thickness and subcortical volume changes,	At baseline patients with PD and probable RBD showed bilateral inferior temporal cortex thinning when compared with patients with PD without RBD Longitudinally, PD with RBD showed a significant increase in the rate of thinning in the left insula compared with the PD without RBD, being the increased thinning correlated with decreased cognitive performance. Patients with probable RBD showed volume decrease over time in the amygdale, pallidum, and left caudate nucleus. The volume changes in the left caudate nucleus were correlated with global cognition.
Jiang et al., 2021 [188]	50 PD patients (26 with probable RBD)/26	3.0 T MRI T1-weighted acquisition; assessment of gray matter alterations with VBM; study of seed-based functional connectivity using resting-state fMRI	Patients with PD and RBD showed, when compared with patients with PD without RBD a relatively low GMV in the right superior occipital gyrus and high GMV in the cerebellar vermis IV/V. Compared with patients with PD without RBD, patients with PD and RBD showed decreased functional connectivity between the right superior occipital gyrus and the posterior regions, including left superior parietal gyrus, left calcarine sulcus, and left fusiform gyrus.
Marques et al., 2021 [189]	17/14	3.0 T MRI T1-weighted acquisition; assessment of grey matter alterations with VBM; study of the whole brain functional connectivity using seed-based analysis of resting-state fMRI	No significant clusters of reduced GMV between iRBD and controls. Compared with controls, iRBD patients showed decreased functional connectivity between the limbic striatum and temporo-occipital regions. The presence of impulse control disorder in iRBD patients was associated with decreased connectivity between the limbic striatum and clusters located in lingual and fusiform gyrus, and cuneus.
Wakasugi et al., 2021 [190]	50/70	3.0 T MRI T1-weighted acquisition; study of the whole brain functional connectivity using resting-state fMRI with GE-EPI	Patients with RBD showed reduced striatal-prefrontal functional connectivity in executive control, suggesting executive dysfunctions, and lack of abnormalities in the default mode network. Patients with RBD showed reduced midbrain-pallidum functional connectivity in the basal ganglia network and reduced motor and somatosensory cortex functional connectivity in the sensorimotor network.
Li et al., 2021 [191]	32/33	3.0 T MRI T1-weighted acquisition; assessment of gray matter alterations with VBM; study of the whole brain functional connectivity using seed-based analysis of resting-state fMRI	Patients with iRBD had significantly reduced GMV in the brainstem, anterior cingulate, and insula compared with controls Patients with iRBD had reduced functional connectivity between the brainstem and the cerebellum posterior lobe, temporal lobe, and anterior cingulated. In patients with iRBD, both reduced GMV and decreased functional connectivity were negatively correlated with the Scales for Outcomes in Parkinson’s Disease-Autonomic scores.
Gan et al., 2021 [192]	126 PD patients (45 with probable RBD)/37	3.0 T MRI T1-weighted acquisition; study of DFC using resting-state fMRI and a sliding-window analysis,	Compared with patients with PD without RBD, patients with PD and RBD showed a brain pattern mainly marked by weaker positive couplings between visual network and default mode network, default mode network and basal ganglia network, and within default mode network. Both PD patients with or without RBD had shorter dwell time and fewer occurrences in State III (by positive correlations between visual and default mode networks, basal ganglia and default mode networks, and positive within-network coupling of sensorimotor network compared to controls).
Zhou et al., 2021 [193]	34/32 (+38 PD patients)	3.0 T MRI T1-weighted acquisition, and processed with DTI; calculation of free-water maps with a bi-tensor model based on the diffusion measurements; assessment of DAT binding with ^18^F-FP-CIT PET	Free-water values in the posterior SN were significantly higher in the patients with iRBD than in controls, but significantly lower than in patients with PD. Posterior SN free-water values were negatively associated with striatal DAT binding ratios in iRBD patients. Longitudinal free-water imaging analyses (in 22 iRBD patients and 18 controls) identified increased mean free-water values in posterior SN of iRBD patients over time.
Zhang et al., 2021 [194]	29/28	3.0 T MRI T1-weighted acquisition, processing with SWI and quantitative susceptibility mapping for evaluation of the nigrosome-1 (N1) sign in the SN, global and regional high-iron content, and volume of subcortical nuclei.	Compared with controls, a higher number of iRBD patients showed N1 loss. iRBD patients showed a reduced volume of the right caudate nucleus. Global and RII iron of the subcortical nuclei was similar in iRBD patients and controls

## Data Availability

Not applicable.
